# Association between air pollutants and birth defects in Xiamen, China

**DOI:** 10.3389/fped.2023.1132885

**Published:** 2023-05-25

**Authors:** Zhimeng Huang, Yue Qiu, Jiawen Qi, Xiaohui Ma, Qiliang Cheng, Jinzhun Wu

**Affiliations:** ^1^ Department of Pediatrics, Women and Children's Hospital, School of Medicine, Xiamen University, Xiamen, China; ^2^ Key Laboratory of Urban Environment and Health, Institute of Urban Environment, Chinese Academy of Sciences, University of Chinese Academy of Sciences, Xiamen, China

**Keywords:** birth defects, air pollution, etiology, China, congenital heart disease

## Abstract

**Objective:**

To explore the relationship between common air pollution and common birth defects, and to provide reference for the prevention of birth defects.

**Methods:**

We conducted a case-control study in Xiamen, a city in southeastern China from 2019 to 2020. Logistics regression was used to analyze the relationship between sulfur dioxide(SO_2_), fine particulate matter 2.5(PM_2.5_), nitrogen dioxide(NO_2_), ozone(O_3_), carbon monoxide(CO) and the occurrence of common birth defects such as congenital heart disease, facial cleft, and finger deformity.

**Results:**

SO_2_ significantly increased the risk of birth defects such as congenital heart disease, cleft lip and/or cleft palate, and ear deformity in the first and second months of pregnancy.

**Conclusion:**

Exposure to common air pollutants increases the risk of birth defects, and SO_2_ significantly affects the occurrence of birth defects in the first two months of pregnancy.

## Introduction

Birth defects also known as congenital malformations or congenital diseases, is due to external environmental factors, genetic factors or both lead to embryonic growth, differentiation in the process of structural, functional, metabolic abnormalities (1). With the advancement of modern medicine, the number of perinatal deaths has decreased by 23.5%, of which 40% are due to infectious diseases, while perinatal deaths caused by congenital malformations have only decreased by 8.4%, indicating the importance of preventing birth defects (2), Children with birth defects are more likely to suffer from neonatal asphyxia, infections and even death in hospital than those without birth defects, research suggests (3). Congenital malformations are the second most common cause of infant death after preterm birth, accounting for at least 20% of infant deaths (4). According to the data released by China’s latest birth defect prevention and control report, the incidence of birth defects in China is close to 5.6% in middle-income developed countries, with an annual increase of about 900,000 cases. The proportion of birth defects in infant deaths has risen from the fourth to the second (5).

About 65%–75% of the causes of birth defects are caused by multiple genes or other mixed factors, of which monogenic diseases account for about 15%–20%, chromosomal abnormalities account for about 5%, and environmental factors account for about 10% (6). Air pollutants such as O_3_, PM_2.5_, PM_10_ can increase the incidence of respiratory infections, asthma, coronary heart disease, birth defects and other diseases, eventually leading to increased mortality (7, 8). Since 2005, Beate Ritz used a case-control study to find that with the increase of CO exposure dose, the incidence of ventricular septal defect increased in a dose-response relationship. Similarly, the risk of aortic valve defects, pulmonary valve malformations, and conotruncal defects also increased with increasing O_3_ exposure (9). The research on air pollution and birth defects is more and more in-depth. For example, Cijiang Yao et al.divided the period of air pollution exposure into four periods: pre-pregnancy and pregnancy, and found that for every 10 μg/m^3^ increase in SO_2_ concentration in the first three months of pregnancy, the risk of birth defects increased by about 20% (10). A population-based case-control study in Taiwan also showed that exposure to pollutants such as O_3_, PM_10_, and SO_2_ during early pregnancy increases the risk of various congenital heart disease subtypes (11).

In this paper, 69,995 cases of neonatal births in Xiamen, a city in southeast China, were counted, and the data of birth defects were analyzed. The general situation of children with birth defects, the relationship between disease order, the occurrence and development trend of air pollution, and the relationship between air pollutants and birth defects were analyzed. It is hoped to help readers understand the occurrence of birth defects and related causes in southeastern China.

## Methods

### Data sources

The clinical case data of this study were derived from a retrospective case-control study conducted in Xiamen City from April 1, 2019 to June 30, 2020. Hospitals diagnosed and reported birth defects according to the requirements of the “China Birth Defects Monitoring Program”. The data of birth defects were extracted from the monitoring platform of birth defects of women and children affiliated to Xiamen University. The birth data of the healthy control group in the same period and the same area were obtained from the electronic medical record system (EMR) of Xiamen University Women’s and Children’s Hospital, including maternal age, parity, nationality, pregnancy disease, infant gestational age, birth weight, gender, birth defect category, and so on (12). This study passed the ethical review of maternal and child health care institutions in Xiamen, and our research was conducted in accordance with local and national regulations.

### Exposure assessment

We collected air pollution data from January 1, 2019 to December 31, 2019. These data were from Xiamen Ecological Environment Bureau. A total of 39 air-quality monitoring stations (AQMS) monitored common air pollutants such as SO_2_, PM_2.5_, NO_2_, O_3_, and CO to obtain hourly average data and calculate monthly average data. We took the last menstrual time as the starting time of exposure (13). For pregnant women with less than 15 days of pregnancy, exposure analysis was performed according to the previous month, which was divided into the first month of pregnancy, the second month of pregnancy, and the third month of pregnancy (14). The geographical coordinates were obtained from the residence of pregnant women. The Kriging interpolation method was used to match the time and place exposure of pollutant values in the first three months of pregnancy, and the spatial location exposure values were obtained (15, 16).

### Exclusion and inclusion criteria

Inclusion criteria: 1. Regular prenatal examination and resident population in Xiamen; 2. Birth defect group was diagnosed by doctors during pregnancy or childbirth with birth defect diseases; 3. The control group was a healthy fetus with full-term normal birth weight, and no abnormalities were found by imaging physicians, obstetricians, and neonatologists.

Exclusion criteria:1. No regular prenatal examination or floating population in Xiamen; 2. There were low birth weight, macrosomia, birth defects in the control group; 3. There are clear genetic diseases in the birth defect group, such as Down syndrome, Turner syndrome or other chromosomal and genetic abnormalities; 4. The duration of pregnancy in the birth defect group did not exceed 3 months.

### Statistical analysis

Descriptive analysis was performed on the occurrence of common air pollutants and common birth defect diseases, and the social demographic characteristics of the birth defect group and the control group were compared by Chi-square test. Mann-Kendall trend test was used to analyze the time trend of air pollutant concentration. Pearson test was used to analyze the correlation between air pollution concentration and birth defects. The variance inflation factor (VIF) was used to verify the collinearity between air pollutants and birth defect diseases. When VIF > 10, it was suggested that there was collinearity and relevant factors could not be included in the analysis (12).

The occurrence of birth defects such as congenital heart disease, finger deformity, and ear deformity was regarded as a binary dependent variable. The exposure concentration of air pollutants such as SO_2_, PM_2.5_, NO_2_, O_3_, and CO was regarded as an independent variable. Factors such as age, nationality, parity, and pregnancy disease were used as covariates. Finally, logistic regression was used to analyze the correlation between common air pollutants and common birth defects. The incidence of birth defects (/ten thousand) = the number of related birth defects/the number of perinatal infants × 10,000. Epidata3.1 was used to collect data, Arcmap10.3 was used to analyze and process air pollution data, and pollutant distribution map was made. R4.04 was used for relevant statistical analysis. Bilateral test was used, and *P* < 0.05 indicated that the difference was statistically significant.

## Results

### General analysis of birth defects

A total of 1,208 children with birth defects were born, with a total of 69,995 births. The incidence of birth defects was 172.58/10,000. The common birth defects were congenital heart disease (86.72/10,000), finger deformity (10.85/10,000), cleft lip and (or) cleft palate (6.85/10,000), ear deformity (6.86/10,000) and other diseases (Table 1). In the case group, there were 686 cases (56.79%) of pregnant women over 30 years old, 923 cases (76.41%) of pregnant women living in urban areas, and 682 cases (56.46%) of male children. Chi-square test *X*^2^ = 4.38, *P* < 0.05, indicating that the difference between fetuses of different genders was statistically significant. There were 824 cases (68.21%) of full-term infants with gestational age greater than 37 weeks. Chi-square test *X*^2 ^= 41.87, *P *< 0.05, indicating that there was statistical significance between different gestational weeks. There were 79 cases (6.51%) of gestational diabetes during pregnancy. Chi-square test X^2^ = 19.5, *P* < 0.05, indicating that gestational diabetes during pregnancy was statistically significant (Table 2).

**Table 1:** Birth defect disease sequence.

Rank	BD^a^	No.	Incidence
1	CHD^b^	607	86.7205
2	Finger deformity	76	10.8579
3	Cleft lip and/or cleft palate	48	6.8576
4	Aural deformity	48	6.8576
5	Congenital hydronephrosis	34	4.8575
6	Chromosome abnormality	31	4.4289
7	G-6-PD^c^	28	4.0003
8	Down syndrome	26	3.7146
9	Foot deformities	22	3.1431
10	Cryptorchidism	21	3.0002

^a^ BD, birth defect.

^b^ CHD, congenital heart disease.

^c^ G-6-PD, Glucose 6 phosphatase deficiency.

**Table 2:** General analysis of birth defects.

		Case (%)	Control (%)	***χ* ^2^**	P*
Location	Village	285 (23.59%)	230 (59.13%)	170.04	<0.05
	City	923 (76.41%)	159 (40.87%)		
Gender	Male	682 (56.46%)	196 (50.39%)	4.38	<0.05
	Female	526 (43.54%)	193 (49.61%)		
Parity	0	65 (5.38%)	140 (35.99%)	264	<0.05
	1	554 (45.86%)	144 (37.02%)		
	2	490 (40.56%)	69 (17.74%)		
	≥3	99 (8.20%)	36 (9.25%)		
Age	<20	13 (1.08%)	3 (0.77%)	22.05	<0.05
	20-24	110 (9.11%)	35 (9.00%)		
	25–29	399 (33.03%)	168 (43.19%)		
	30–34	458 (37.91%)	144 (37.02%)		
	35–39	185 (15.31%)	32 (8.23%)		
	≥40	43 (3.56%)	7 (1.80%)		
Race	Han	1165 (96.44%)	376 (96.66%)	0.041	>0.05
	Non-ha	43 (3.56%)	13 (3.34%)		
Gestational weeks	<37	384 (31.79%)	58 (14.91%)	41.87	<0.05
	≥37	824 (68.21%)	331 (85.09%)		
Season of pregnancy	Spring	311 (25.75%)	101 (25.96%)	2.46	>0.05
	Summer	326 (26.99%)	95 (24.42%)		
	Autumn	288 (23.84%)	88 (22.62%)		
	Winter	283 (23.43%)	105 (26.99%)		
Gestational hypertension	Yes	16 (1.35%)	7 (1.87%)	1.158	>0.05
	No	1192 (98.65%)	382 (98.13%)		
Gestational diabete	Yes	79 (6.51%)	45 (11.52%)	19.5	<0.05
	No	1113 (92.15%)	344 (88.48%)		
Hepatitis b	Yes	115 (9.5%)	37 (9.57%)	0.004	>0.05
	No	1093 (90.5%)	352 (90.43%)		
Allergic history	Yes	56 (4.64%)	10 (2.47%)	9.179	<0.05
	No	1152 (95.36%)	379 (97.53%)		
Pregnant history	Yes	808 (66.87%)	239 (61.48%)	8.432	<0.05
	No	400 (33.13%)	150 (38.52%)		
Adverse pregnancy	Yes	554 (45.85%)	151 (38.82%)	13.536	<0.05
	No	654 (54.15%)	238 (61.18%)		
Reproductive system surgery	Yes	578 (47.87%)	90 (23.04%)	180.131	<0.05
	No	630 (52.13%)	299 (76.96%)		

^*^ P, statistically significant result a result at ***P*** < 0.05.

### General analysis of air pollutants

**Figure 1:**
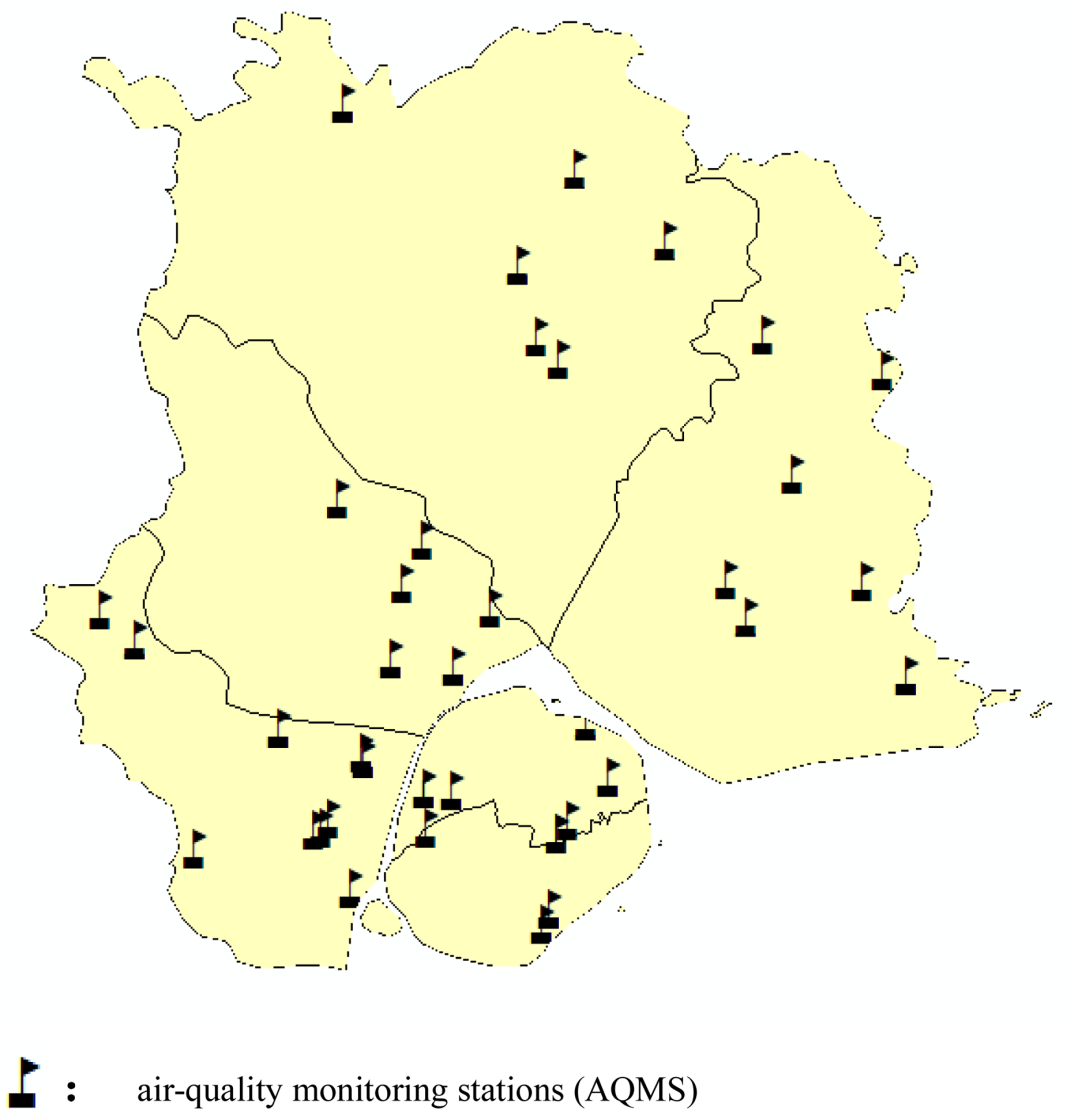
Location of air-quality monitoring station.

A total of 39 air-quality monitoring stations (AQMS) in Xiamen City monitored the real-time changes of air pollutant concentrations such as SO_2_, NO_2_, O_3_, PM_2.5_ and CO from January 1, 2019 to December 31, 2019 (Figure 1). It can be seen from Table 3 that the average concentration of air pollutants in different months is different. The average concentration of SO_2_ reaches the maximum in March, which is 11.19 ug/m^3^, and the concentration reaches the minimum in February, which is 5.03 ug/m^3^. The results of variance analysis show that *F* = 4.214, *P* < 0.05, indicating that there are statistical differences between the concentrations in different months, and the concentration of SO_2_ varies in different months. The trend test of air pollutant concentration showed that *Z* = −0.62, *P* > 0.05, indicating that there was no downward trend in concentration between different months (Table 3).

**Table 3:** Air pollutants in general.

(ug/m3)	.1mon	.2 mon	.3 mon	.4 mon	.5 mon	.6 mon	.7 mon	.8 mon	.9 mon	.10 mon	.11 mon	.12 mon	**Z**	Pz*	**F**	Pf*
SO2	8.052	5.659	11.195	6.634	6.486	5.114	5.033	5.336	5.489	6.473	6.433	7.431	−0.617	0.537	4.214	<0.05
NO2	32.125	21.943	32.903	30.546	25.035	19.649	16.768	16.831	17.314	19.332	23.626	31.595	−1.029	0.304	21.466	<0.05
O3	52.375	50.503	58.780	56.992	63.414	45.193	40.904	49.319	72.034	86.091	73.322	52.765	1.029	0.304	41.794	<0.05
PM2.5	29.373	25.215	28.521	26.604	22.407	14.688	14.266	14.815	17.136	25.092	21.021	26.317	−1.440	0.150	55.163	<0.05
CO	643.673	614.269	565.603	542.613	445.862	404.368	380.624	413.717	430.594	517.542	450.286	539.343	−1.166	0.244	25.965	<0.05

^*^ Pz, statistically significant result a result at *P* < 0.05.

^*^ Pf, statistically significant result a result at *P* < 0.05.

Descriptive analysis of the concentration of air pollutants in the birth defect group and the control group showed that the mean concentrations of SO_2_ and O_3_ in the birth defect group were 5.88 ug/m^3^ and 58.46 ug/m^3^, respectively, which were higher than those in the healthy control group. The mean concentrations of NO_2_, PM_2.5_ and CO in the control group were higher than those in the birth defect group. The rank sum test of SO_2_ concentration in the birth defect group and the control group showed that *Z* = −0.87, *P *= 0.38, the difference was not statistically significant. Other pollutants *P* < 0.05, indicating that the difference in the concentration of O_3_, NO_2_, CO and PM_2.5_ between the birth defect group and the control group was statistically significant (Table 4).

**Table 4:** Air pollutant concentration distribution.

Air pollution	(μg/m^3^)	BD	Controls	AQMS	***Z***	*P*
SO_2_	Mean ± SD	5.88 ± 1.31	5.86 ± 1.32	6.09 ± 2.30	−0.87	0.38
	Median	5.82	5.76	5.80		
	Range	2.96–15.97	3.29–14.51	1.39–19.74		
	IQR	4.94–6.51	4.85–6.52	4.42–7.23		
NO_2_	Mean ± SD	24.73 ± 7.01	26.49 ± 7.73	24.16 ± 10.64	−6.55	<0.05
	Median	22.59	24.81	22.61		
	Range	8.29–52.24	9.14–48.67	1.01–61.87		
	IQR	19.76–29.49	20.15–32.94	16.99–30.41		
O_3_	Mean ± SD	58.46 ± 14.97	52.35 ± 16.12	58.29 ± 17.84	−8.91	<0.05
	Median	54.62	51.51	56.47		
	Range	18.11–107.56	3.86–106.29	5.30–109.25		
	IQR	47.66–66.82	46.20–59.22	47.25–69.68		
PM_2.5_	Mean ± SD	21.39 ± 5.94	23.29 ± 6.42	22.21 ± 7.08	−9.50	<0.05
	Median	21.72	24.58	21.84		
	Range	10.4–45.18	10.35–42.32	9.16–46.82		
	IQR	15.83–26.01	16.62–28.13	16.78–26.76		
CO	Mean ± SD	494.75 ± 91.80	526.97 ± 90.13	499.96 ± 132.04	−10.15	<0.05
	Median	494.75	533.42	500.42		
	Range	274.56–771.80	313.13–725.96	192.17–933.11		
	IQR	428.48–568.06	453.72–605.12	403.90–584.53		

BD, birth defect; SD, standard deviation; IQR, interquartile range; AQMS, air-quality monitoring stations.

### Correlation analysis between air pollution and birth defects

Pearson correlation analysis was performed on common air pollutants and birth defect diseases. The results showed that SO_2_ was strongly positively correlated with NO_2_, SO_2_ was positively correlated with PM_2.5_ and CO, NO_2_ was strongly positively correlated with PM_2.5_, NO_2_ was positively correlated with CO, and PM_2.5_ was strongly positively correlated with CO (Table 5).

**Table 5:** Correlation analysis between air pollution and birth defects.

	SO2	NO2	O3	PM2.5	CO	BDs’ Number^†^	BDs’ incidence^‡^
SO2	1	0.818**	0.173	0.736*	0.585*	−0.202	−0.216
NO2	0.818**	1	−0.014	0.849**	0.733*	0.171	0.151
O3	0.173	−0.014	1	0.333	0.138	0.051	−0.138
PM2.5	0.736*	0.849**	0.333	1	0.919**	0.215	0.177
CO	0.585*	0.733*	0.138	0.919**	1	0.306	0.308
BDs’ Number^†^	−0.202	0.171	0.051	0.215	0.306	1	0.958**
BDs’ incidence^‡^	−0.216	0.151	−0.138	0.177	0.308	0.958**	1

^†^ BDs’ Number: birth defect Number.

^‡^ BDs’ incidence: birth defect incidence.

*P < 0.05; **P < 0.01

### Colinearity analysis of air pollution and birth defects

The results of collinearity analysis between air pollutants and birth defect diseases showed that there was no collinearity between birth defect diseases and air pollution such as SO_2_, NO_2_, O_3_, PM_2.5_ and CO, and VIF was less than 10, so they could be included in the single pollutant model respectively. The defect diseases and SO_2_, NO_2,_ O_3_, PM_2.5_, CO and other air pollution are analyzed together. The results show that PM_2.5_ has collinearity in the first month of pregnancy and the second month of pregnancy, and VIF is equal to 12.62 and 13.35 respectively. Therefore, PM_2.5_ is not included in the co-pollutant model (Table 6).

**Table 6:** The collinearity analysis of air pollution and birth defects.

	Single-pollutant model	Multi-pollutant models	VIF^a^
First month SO2	1.735	6.808	<10
Second month SO2	2.195	6.614	<10
Third month SO2	2.066	4.189	<10
First month O3	2.277	4.909	<10
Second month O3	3.468	7.836	<10
Third month O3	1.881	3.050	<10
First month PM2.5	2.122	**12** **.** **625**	>10
Second month PM2.5	3.060	**13**.**350**	>10
Third month PM2.5	1.923	9.699	<10
First month CO	2.678	6.653	<10
Second month CO	3.037	5.710	<10
Third month CO	1.863	4.847	<10
First month NO2	1.312	5.932	<10
Second month NO2	1.652	4.656	<10
Third month NO2	1.491	7.476	<10

Font bolding hints the existence of meaning

^a^ VIF, variance inflation factor.

### Common air pollution and birth defects

#### Single pollutant model of air pollutants and birth defect diseases

All birth defect diseases, congenital heart disease, finger deformity, cleft lip and (or) cleft palate, external ear deformity and air pollution such as SO_2_, NO_2_, O_3_, PM_2.5_ and CO were analyzed by logistics regression analysis of single pollutants in the first three months of pregnancy. The results showed that SO_2_, NO_2_, O_3_, PM_2.5_ and CO had positive effects on different birth defect diseases in the first three months of pregnancy, among which SO_2_ had the greatest impact on various birth defect diseases.

SO_2_ during the first and (or) second trimesters of pregnancy increases the incidence of all birth defects, congenital heart disease, cleft lip and (or) palate, and external ear malformations. In the first month of pregnancy and the second month of pregnancy, every 10ug/m^3^ increase in SO_2_ will lead to an increase of 21%, 35% and 31%, 33% in all birth defects and congenital heart disease, respectively. In the second month of pregnancy, for every 10 ug/m^3^ increase in SO_2_, the cOR of cleft lip and (or) cleft palate was 1.37,95% CI = (1.00,1.89). The maternal age, nationality, parity, pregnancy disease and other factors were brought into the analysis as covariates, and aOR = 1.498,95% CI = (1.062,2.113). In the first month of pregnancy, for every 10 ug/m^3^ increase in SO_2_, the incidence of ear malformations increased by 65%, and aOR = 1.613 after covariate adjustment, the change was not significant. The remaining pollutants CO, NO_2_, O_3_ and PM_2.5_ will have positive effects on different birth defect diseases in the first three months of pregnancy, but have little effect on birth defect diseases (Tables 7, 8).

**Table 7:** Single pollutant model of air pollutants and birth defects.

Single-pollutant model		SO2		O3		PM2.5	
BD	Exposure window	cOR (95% CI)	aOR (95% CI)	cOR (95% CI)	aOR (95% CI)	cOR (95% CI)	aOR (95% CI)
All BD	First month	**1.21** **(1.08**, **1.36)**	**1.34** **(1.18, 1.52)**	**1.01** **(1.00, 1.03)**	1.009 (0.994, 1.024)	**1.04** **(1.07, 1.01)**	**1.05** **(1.02, 1.09)**
	Second month	**1.35** **(1.17, 1.54)**	**1.46** **(1.25,1.70)**	**1.02** **(1.00, 1.03)**	**1.020** **(1.003, 1.037)**	1.01 (0.98, 1.05)	1.03 (0.99, 1.07)
	Third month	0.91 (0.80, 1.04)	0.94 (0.81, 1.09)	1.01 (0.99, 1.02)	1.012 (0.999, 1.024)	**1.04** **(1.07, 1.02)**	**1.04** **(1.01, 1.07)**
CHD	First month	**1.31** **(1.15, 1.50)**	**1.40** **(1.21, 1.63)**	1.01 (0.99, 1.02)	0.997 (0.981, 1.014)	**1.04** **(1.01, 1.08)**	**1.05** **(1.02, 1.09)**
	Second month	**1.33** **(1.15, 1.55)**	**1.42** **(1.69, 1.20)**	**1.02** **(1.00, 1.04 )**	**1.024** **(1.005, 1.043)**	1.02 (0.98, 1.07)	1.03 (0.99, 1.08)
	Third month	0.92 (0.80, 1.06)	0.95 (0.80, 1.12)	1.01 (0.99, 1.02 )	1.012 (0.998, 1.027)	**1.06** **(1.03, 1.09)**	**1.04** **(1.01, 1.08)**
Finger deformity	First month	0.83 (0.65, 1.06)	0.89 (0.69, 1.15)	**1.03** **(1.00, 1.06)**	0.974 (0.946, 1.003)	0.99 (0.94, 1.05)	0.997 (0.944, 1.054)
	Second month	1.16 (0.88, 1.54)	1.26 (0.93, 1.72)	1.02 (0.99, 1.05)	1.015 (0.986, 1.045)	0.97 (0.89, 1.05)	0.97 (0.891, 1.055)
	Third month	1.08 (0.80, 1.45)	1.00 (0.73, 1.37)	**1.03** **(1.01, 1.06)**	**1.035** **(1.010, 1.060)**	0.96 (0.89, 1.02)	0.949 (0.887, 1.017)
Cleft lip and(or) cleft palate	First month	1.11 (0.84, 1.48)	1.221 (0.904, 1.651)	**1.04** **(1.01, 1.07)**	**1.037** **(1.004, 1.071)**	0.99 (0.92, 1.06)	0.999 (0.928, 1.076)
	Second month	**1.37** **(1.00, 1.89)**	**1.498** **(1.062, 2.113)**	0.99 (0.96, 1.03)	0.996 (0.962, 1.030)	0.95 (0.86, 1.05)	0.944 (0.855, 1.043)
	Third month	0.72 (0.50, 1.05)	**1.550** **(1.029, 2.336)**	1.02 (0.99, 1.05)	1.015 (0.983, 1.047)	1.02 (0.95, 1.10)	1.017 (0.939, 1.102 )
Aural deformity	First month	**1.65** **(1.19, 2.28)**	**1.613** **(1.134, 2.294)**	0.99 (0.96, 1.03)	0.989 (0.951, 1.029)	**1.08** **(1.00, 1.16)**	0.943 (0.874, 1.017)
	Second month	1.03 (0.71, 1.49)	1.073 (0.707, 1.628)	1.03 (0.99, 1.07)	1.034 (0.993, 1.076)	1.05 (0.96, 1.15)	1.054 (0.959, 1.160)
	Third month	1.21 (0.84, 1.76)	1.150 (0.781, 1.692)	1.00 (0.97, 1.03)	1.001 (0.970, 1.033)	0.99 (0.92, 1.07)	0.989 (0.911, 1.073)

cOR^a^, Unadjusted odds ratio; aOR^b^, Adjusted odds ratios for age, gender, medication history, family history, and so on; 95% CI^c^, 95% confidence interval; BD^a^, birth defect; CHD^b^, congenital heart disease.

Font bolding hints the existence of meaning

**Table 8:** Single pollutant model of air pollutants and birth defects.

Single-pollutant model		CO		NO2	
	Exposure window	cOR (95% CI)	aOR (95% CI)	cOR (95% CI)	aOR (95% CI)
	First month	0.99 (0.99, 1.01)	0.999 (0.997, 1.001)	**1.04** **(1.02, 1.06)**	**1.057** **(1.036, 1.079)**
All BD	Second month	**1.01** **(1.01, 1.00)**	**1.004** **(1.002, 1.007)**	**1.03** **(1.00, 1.05)**	**1.041** **(1.015, 1.067)**
	Third month	1.00 (0.99, 1.00)	1.001 (0.999, 1.003)	**1.04** **(1.02, 1.06)**	**1.034** **(1.012,1.056)**
	First month	0.999 (0.997, 1.002)	0.999 (0.997, 1.002)	**1.03** **(1.01, 1.05)**	**1.050** **(1.027, 1.075)**
CHD	Second month	**1.004** **(1.002, 1.007)**	**1.004** **(1.001, 1.007)**	**1.04** **(1.01, 1.06)**	**1.042** **(1.013, 1.073)**
	Third month	0.999 (0.997, 1.001)	1.000 (0.998, 1.003)	**1.05** **(1.03, 1.07)**	**1.041** **(1.015, 1.067)**
	First month	**1.004** **(1.001, 1.008)**	**1.004** **(1.000, 1.008)**	1.007 (0.998, 1.016)	**1.01** **(1.00, 1.02)**
finger deformity	Second month	0.999 (0.994, 1.003)	0.998 (0.992, 1.004)	0.993 (0.946, 1.043)	1.00 (0.95, 1.05)
	Third month	0.999 (0.994, 1.004)	0.999 (0.994, 1.004)	**1.074** **(1.124, 1.026)**	**1.08** **(1.03, 1.14)**
	First month	1.001 (0.996, 1.007)	1.002 (0.997, 1.008)	**1.07** **(1.02, 1.12)**	**1.062** **(1.009, 1.116)**
Cleft lip and(or) cleft palate	Second month	**1.006** **(1.000, 1.012)**	**1.007** **(1.001, 1.014)**	1.04 (0.98, 1.10)	1.045 (0.983, 1.110)
	Third month	1.001 (0.995, 1.006)	1.001 (0.995, 1.007)	0.99 (0.93, 1.04)	0.977 (0.920, 1.036)
	First month	0.998 (0.993, 1.004)	0.999 (0.993, 1.006)	**1.08** **(1.03, 1.13)**	**1.071** **(1.016, 1.127)**
aural deformity	Second month	0.997 (0.991, 1.003)	0.997 (0.990, 1.004)	1.03 (0.97, 1.09)	1.038 (0.975, 1.105)
	Third month	1.002 (0.997, 1.008)	1.002 (0.996, 1.008)	1.02 (0.96, 1.08)	1.008 (0.946, 1.074)

cOR^a^, Unadjusted odds ratio; aOR^b^, Adjusted odds ratios for age, gender, medication history, family history, and so on; 95% CI^c^, 95% confidence interval; BD^a^, birth defect; CHD^b^, congenital heart disease.

Font bolding hints the existence of meaning

#### Multi-pollutant model of air pollutants and birth defects

In the multi-pollutant model, it can be seen from the above that PM_2.5_ has collinearity with other pollutants in the first two months of pregnancy, so PM_2.5_ is not included in the co-pollutant model. The results showed that SO_2_, CO, NO_2_ and O_3_ had different effects on all birth defects, congenital heart disease, cleft lip and (or) cleft palate, finger deformity and external ear deformity in the first three months of pregnancy. Among them, SO_2_ has a greater impact on the occurrence of cleft lip and (or) cleft palate and external ear malformations in the second month of pregnancy. For every 10 ug/m^3^ increase in SO_2_, the occurrence of cleft lip and/or cleft palate and external ear malformations will increase by 73% and 150%, respectively. After covariate adjustment, aOR was 1.968 and 2.347, respectively. The remaining pollutants CO, NO_2_ and O_3_ will have positive effects on different birth defect diseases in the first three months of pregnancy, but have little effect on birth defect diseases (Tables 9, 10).

**Table 9:** Multi-pollutant model of air pollutants and birth defects.

Multi-pollutant models		SO2		O3	
	Exposure window	cOR (95% CI)	aOR (95% CI)	cOR (95% CI)	aOR (95% CI)
	First month	0.959 (0.774, 1.188)	0.887 (0.699, 1.125)	**1.02** **(1.003, 1.038)**	**1.02** **(1.001, 1.039)**
All BD	Second month	0.985 (0.782, 1.241)	1.116 (0.862, 1.445)	1.013 (0.993, 1.033)	1.004 (0.982, 1.026)
	Third month	1.134 (0.936, 1.373)	1.11 (0.899, 1.37)	**1.017** **(1.002, 1.032)**	**1.027** **(1.01, 1.043)**
	First month	0.845 (0.655, 1.092)	0.858 (0.644, 1.142)	1.018 (0.998, 1.038)	1.01 (0.988, 1.033)
CHD	Second month	1.036 (0.798, 1.346)	1.12 (0.836, 1.501)	1.011 (0.988, 1.033)	1.009 (0.984, 1.035)
	Third month	1.209 (0.98, 1.491)	1.131 (0.887, 1.442)	**1.019** **(1.002, 1.036)**	**1.028** **(1.009, 1.046)**
	First month	0.760 (0.463, 1.246)	0.754 (0.415, 1.368)	1.000 (0.960, 1.041)	1.006 (0.964, 1.049)
Finger deformity	Second month	1.140 (0.696, 1.867)	1.469 (0.829, 2.604)	0.994 (0.955, 1.035)	0.983 (0.942, 1.026)
	Third month	1.460 (0.988, 2.158)	1.267 (0.826, 1.943)	**1.043** **(1.014, 1.073)**	**1.051** **(1.021, 083)**
	First month	**1.730** **(1.042, 2.874)**	**1.968** **(1.142, 3.392)**	1.018 (0.975, 1.064)	1.021 (0.975, 1.069)
Cleft lip and(or) cleft palate	Second month	0.763 (0.418, 1.392)	0.843 (0.456, 1.559)	1.000 (0.954, 1.048)	0.999 (0.952, 1.047)
	Third month	0.939 (0.572, 1.541)	0.784 (0.452, 1.359)	**1.046** **(1.004, 1.089)**	**1.047** **(1.003, 1.093)**
	First month	1.095 (0.546, 2.195)	1.089 (0.499, 2.375)	0.987 (0.94, 1.036)	0.982 (0.929, 1.038)
Aural deformity	Second month	**2.506** **(1.069, 5.848)**	2.347 (0.957, 5.747)	1.051 (0.994, 1.111)	1.056 (0.991, 1.124)
	Third month	1.489 (0.904, 2.45)	1.264 (0.717, 2.227)	0.995 (0.959, 1.032)	1.000 (0.959, 1.042)

cOR^a^, Unadjusted odds ratio; aOR^b^, Adjusted odds ratios for age, gender, medication history, family history, and so on; 95%CI^c^, 95% confidence interval; BD^a^, birth defect; CHD^b^, congenital heart disease.

Font bolding hints the existence of meaning

**Table 10:** Multi-pollutant model of air pollutants and birth defects.

Multi-pollutant models		CO		NO2	
	Exposure window	cOR (95% CI)	aOR (95% CI)	cOR (95% CI)	aOR (95% CI)
	First month	1.002 (0.999, 1.005)	1.003 (0.999, 1.006)	0.994 (0.963, 1.025)	0.986 (0.953, 1.02)
All BD	Second month	**1.007** **(1.004, 1.010)**	**1.008** **(1.005, 1.011)**	1.025 (0.992, 1.059)	1.029 (0.991, 1.067)
	Third month	0.999 (0.996, 1.002)	1.000 (0.997, 1.003)	0.98 (0.948, 1.012)	0.984 (0.95, 1.019)
	First month	1.002 (0.999, 1.005)	1.003 (0.999, 1.006)	1.011 (0.976, 1.049)	0.996 (0.956, 1.037)
CHD	Second month	**1.008** **(1.011, 1.005)**	**1.008** **(1.012, 1.005)**	1.026 (0.988, 1.065)	1.027 (0.984, 1.073)
	Third month	0.999 (0.996, 1.002)	0.999 (0.995, 1.003)	0.98 (0.945, 1.017)	0.99 (0.95, 1.032)
	First month	1.003 (0.997, 1.01)	1.001 (0.994, 1.008)	1.019 (0.974, 1.067)	1.046 (0.964, 1.136)
Finger deformity	Second month	**1.009** **(1.002, 1.015)**	**1.009** **(1.015, 1.002)**	1.000 (0.924, 1.083)	1.004 (0.917, 1.099)
	Third month	0.998 (0.992, 1.005)	1.001 (0.994, 1.009)	0.964 (0.897, 1.037)	0.946 (0.874, 1.024)
	First month	1.004 (0.995, 1.012)	1.002 (0.993, 1.012)	**1.092** **(1.008, 1.183)**	0.922 (0.847, 1.004)
Cleft lip and(or) cleft palate	Second month	**1.012** **(1.004, 1.019)**	**1.012** **(1.004, 1.020)**	**1.133** **(1.027, 1.250)**	**1.157** **(1.04, 1.286)**
	Third month	0.999 (0.992, 1.007)	1.001 (0.993, 1.009)	0.992 (0.912, 1.079)	0.972 (0.887, 1.065)
	First month	1.004 (0.996, 1.012)	1.004 (0.995, 1.013)	0.917 (0.838, 1.004)	0.933 (0.845, 1.03)
Aural deformity	Second month	0.994 (0.986, 1.002)	0.993 (0.984, 1.002)	1.099 (0.986, 1.225)	1.113 (0.99, 1.251)
	Third month	1.000 (0.992, 1.007)	1.002 (0.994, 1.01)	0.979 (0.894, 1.071)	0.965 (0.872, 1.068)

cOR^a^, Unadjusted odds ratio; aOR^b^, Adjusted odds ratios for age, gender, medication history, family history, and so on; 95% CI^c^, 95% confidence interval; BD^a^, birth defect; CHD^b^, congenital heart disease.

Font bolding hints the existence of meaning

## Discussion

The occurrence of birth defects is affected by many factors, such as advanced maternal age, diabetes during pregnancy, or the use of pregnancy-related drugs, which may lead to an increase in the incidence of birth defects (17, 18). Early in pregnancy, especially at 1–10 weeks of gestation is a critical period of embryonic differentiation (19). Therefore, we chose the first three months of pregnancy to study air pollution and birth defects. This study shows that common air pollutants can lead to an increase in the incidence of birth defects during this period, especially SO_2_ is a high-risk factor leading to an increase in the incidence of common birth defects. In 2016, the results of the global death factor survey showed that air pollutants were the sixth leading cause of death, and 7.5% of global deaths were attributed to ambient air pollution. The countries with higher deaths included China and India (20).

By comparing the particle pollution levels on both sides of the placenta under different particle pollutant exposure levels of pregnant women, it was found that particle pollutants could accumulate on the side of the fetus through the placenta (21). Related mouse embryo experiments show that air pollutants can affect long non-coding RNAs (*lncRNAs*) expression, mitosis, cell respiration, glycolic acid metabolism and proteasome and other biological processes, leading to congenital spinal cord defects (22, 23). There are also related clinical trials found that air pollutants and *ABCC_2_*, *CYP_1_A_1_*, *CYP_2_B_6_*, *CYP_2_C_19_* and other genes between the gene-environment interaction effect, or by up-regulating miRNA to reduce cardiovascular-related gene expression, leading to abnormal expression of related genes, leading to the occurrence of congenital heart disease (24, 25).

Associated mate analyzed the relationship between air pollution and congenital heart disease and found that the more polluted countries have a higher percentage of methylenetetrahydrofolate (*MTHFR*) gene polymorphisms, the higher the risk of congenital heart disease (26). Ahmed Karoui et al. studied mice exposed to NO_2_ and found that NO_2_ exposure impairs mitochondrial function in the heart, which in turn leads to endothelial dysfunction in the coronary arteries and, ultimately, myocardial ischemia (27). A clinical cohort study in Henan Province, China, showed that air pollutants may increase the methylation level of the superoxide dismutase 2 (*SOD_2_*) promoter in umbilical cord blood, leading to adverse pregnancy outcomes (28).

The results of a case-control study in Liaoning Province of China showed that exposure to SO2 increased the incidence of cleft lip and/or cleft palate in the first three months of pregnancy and the first month of pregnancy. The cOR = 1.63,95% CI = (1.38,1.93) of cleft lip and (or) cleft palate in the first month of pregnancy was the same as our results (29). A case-control study in Texas, the United States, showed that PM_2.5_ and SO_2_ increased the incidence of congenital heart disease during the third to eighth weeks of pregnancy, which is the same as our findings in the second month of pregnancy (30). Shuang Zhang et al. used logistic regression to analyze the relationship between SO_2_ and hypospadias. The results showed that SO_2_ increased the risk of hypospadias in the multi-pollutant model in the first month of pregnancy (31). Wang ling ling et al. used the generalized additive model to study the relationship between common air pollutants and birth defects. The results showed that NO_2_ had an impact on all birth defects in the first three months of pregnancy, and the third month of pregnancy had the greatest impact. For every 10 μg/m^3^ increase in NO_2_ concentration, the risk of birth defects increased by 10.3%, which was the same as our conclusion (32).

Ying Zhou et al., conducted a retrospective case-control study on birth defects data in four states of the United States. The results showed that for each quartile increase in NO_2_ concentration, the aOR and 95%CI was 1.15 (1.00,1.32), while O_3_ did not have this relationship (33). Mariam S. Girguis et al. used the generalized additive linear model to study the relationship between traffic-related pollutants and cleft lip and palate, congenital heart disease, and neural tube defects. The results showed that PM_2.5_ was positively correlated with various subtypes of congenital heart disease and negatively correlated with cleft lip and palate. Our results are different from that, considering the different exposure methods of early air pollution (34). A retrospective control study in Ohio, the United States, showed that in the first month of pregnancy, for every 10 μg/m^3^ increase in PM_2.5_ concentration, the aOR and 95% confidence interval of birth defect disease were 1.09 (1.01,1.18), which was the same as our conclusion (35).

Many scientists have studied whether the change of address during pregnancy affects the accuracy of the results. A study of more than 9,000 pregnant women in Connecticut showed that about 11% of pregnant women moved during pregnancy, but the difference in exposure to pollutants was not statistically significant (36). A New York State cohort study similarly showed that a mother’s address at birth can be a good substitute for a mother’s address during pregnancy, with a higher level of consistency observed (37). But a cohort study in China’s Gansu province found that those who moved were less likely to have adverse birth consequences than those who did not, possibly related to reducing environmental harm. Residential mobility should be considered in future environmental exposure assessment studies, as well as the impact of exposure misclassifications and differences among different populations, depending on where you live at birth (38). A mate analysis of environmental exposure during pregnancy and residential mobility during pregnancy showed that mobility was the highest in the second trimester of pregnancy, and mobility usually decreased with age and socioeconomic status, but there were differences among different races and customs. Thus, there may be misclassifications in the environmental exposure estimated from incomplete residential information, but most of the shifts are short-distance, so limited residential data can be used to estimate environmental exposure during pregnancy (39).

### Importance and limitations

For how to reduce the occurrence of birth defect diseases, it is necessary to professionalize birth defect diseases, train specialized nurses to inquire about prenatal exposure factors, and use relevant scales by professional doctors to conduct preliminary classification of diseases, reclassification of disease severity, and research and evaluation of each different phenotype (40, 41).

First of all, this study was diagnosed and classified by professional medical staff according to strict standards, and examined and analyzed by relevant statisticians. Second, this study has a large sample size and a wide coverage area, covering most birth defect diseases, including all cases of live births and stillbirths. It is conducive to the evaluation of risk factors and improves the reliability and representativeness of this study. Thirdly, taking the past history, medication history and family history of the mother and the child as covariates, the adjustment was made to reduce the influence of interference factors. Fourth, there are many air-quality monitoring stations (AQMS) in this paper, the monitoring time is long, and the coverage area is wide, which provides the accuracy of the air pollution value exposed by the children. Fifth, this study covers the relationship between multiple pollutants and multiple birth defects. Finally, during the novel coronavirus epidemic in this study, according to the regional isolation measures, pregnant women spend most of their time resting at home and are forced to wear masks in public places, which increases the rationality of the location exposure matching of air pollution data in this paper (42, 43).

Our study is based on data from monitoring sites, but most of the time maternal rest at home, studies have shown that indoor air pollutants such as smoking, coal, mold exposure can also increase the risk of birth defects, premature birth, low birth weight, which will affect the accuracy of the results (44–47). The main ethnic group in southern China is East Asian yellow people, and the vast majority of pregnant women are Han nationality. There is a lack of assessment of ethnic minority risk factors and different ethnic groups. Finally, this study is a retrospective study, there is a certain memory bias.

## Data Availability

The raw data supporting the conclusions of this article will be made available by the authors, without undue reservation.
